# *In vitro* Chondrocyte Responses in Mg-doped Wollastonite/Hydrogel Composite Scaffolds for Osteochondral Interface Regeneration

**DOI:** 10.1038/s41598-018-36200-x

**Published:** 2018-12-17

**Authors:** Xinning Yu, Tengfei Zhao, Yiying Qi, Jianyang Luo, Jinghua Fang, Xianyan Yang, Xiaonan Liu, Tengjing Xu, Quanming Yang, Zhongru Gou, Xuesong Dai

**Affiliations:** 1 Department of Orthopaedic Surgery, Second Affiliated Hospital, School of Medicine, Zhejiang University, Hangzhou, 310009 China; 2 Orthopaedics Research Institute, Zhejiang University, Hangzhou, 310009 China; 3 Bio-nanomaterials and Regenerative Medicine Research Division, Zhejiang-California International NanoSystems Institute, Zhejiang University, Hangzhou, 310058 China; 4 Department of Orthopaedic Surgery, Hangzhou Mingzhou Hospital (International Medical Center, Second Affiliated Hospital, Zhejiang University), Hangzhou, 311215 China

## Abstract

The zone of calcified cartilage (ZCC) is the mineralized region between the hyaline cartilage and subchondral bone and is critical in cartilage repair. A new non-stoichiometric calcium silicate (10% Ca substituted by Mg; CSi-Mg10) has been demonstrated to be highly bioactive in an osteogenic environment *in vivo*. This study is aimed to systematically evaluate the potential to regenerate osteochondral interface with different amount of Ca-Mg silicate in hydrogel-based scaffolds, and to compare with the scaffolds containing conventional Ca-phosphate biomaterials. Hydrogel-based porous scaffolds combined with 0–6% CSi-Mg10, 6% β-tricalcium phosphate (β-TCP) or 6% nanohydroxyapatite (nHAp) were made with three-dimensional (3D) printing. An increase in CSi-Mg10 content is desirable for promoting the hypertrophy and mineralization of chondrocytes, as well as cell proliferation and matrix deposition. Osteogenic and chondrogenic induction were both up-regulated in a dose-dependent manner. In comparison with the scaffolds containing 6% β-TCP or nHAp, human deep zone chondrocytes (hDZCs) seeded on CSi-Mg10 scaffold of equivalent concentration exhibited higher mineralization. It is noteworthy that the hDZCs in the 6% CSi-Mg10 scaffolds maintained a higher expression of the calcified cartilage zone specific extracellular matrix marker and hypertrophic marker, collagen type X. Immunohistochemical and Alizarin Red staining reconfirmed these findings. The study demonstrated that hydrogel-based hybrid scaffolds containing 6% CSi-Mg10 are particularly desirable for inducing the formation of calcified cartilage.

## Introduction

An osteochondral defect is common, especially in athletes, and this defect is often concomitant with subchondral bone injury^1^. However, current surgical interventions are prone to causing suboptimal clinical outcomes due to donor site morbidity, poor integration, and/or formation of fibrocartilage^2,3^.

The zone of calcified cartilage (ZCC) is a mineralized region in between the hyaline cartilage and subchondral bone. The ZCC serves as a physical barrier and an osteochondral interface to transmit forces. Hunziker *et al*. reported that such a barrier facilitates the formation and maintains the integrity of newly formed cartilage by suppressing the upgrowth of blood vessels into the cartilage compartment and ectopic mineralization^4^. Therefore, regeneration of the calcified cartilage layer is a prerequisite for functional and integrative cartilage repair. The ideal osteochondral interface scaffold must support viability and hypertrophy of the chondrocytes and promote the formation of a calcified cartilage matrix^5^.

Tissue engineering has now emerged as one of the promising alternatives for human tissue or organ repair. Scaffolding is a key component of tissue engineering. It is imperative that the materials with which the construct is made resemble the tissue it replaces^6^. Due to their compositional similarity to natural bone minerals, the synthesized calcium phosphates (CaPs), such as hydroxyapatite (HAp) and β-tricalcium phosphate (β-TCP), have been applied in clinic and osteochondral interface tissue engineering^7,8^. However, lack of osteoinductivity is the major drawback for these materials^9,10^.

Wollastonite (CaSiO_3_; CSi) has been used as an alloy coating, as granules, or as a sintered porous body due to its higher osteoconductivity. Compared with the clinically used bone CaPs implants, wollastonite exhibits higher bioactivity and osteogenesis due to the release of Ca^2+^ and SiO_3_^2−^ ions^11,12^. Magnesium (Mg) ranks fourth among intracellular elements and is crucial to bone mineralization^13^. Recently we developed a series of nonstoichiometric wollastonite materials via dilute Mg substitution of Ca (CSi-Mg) which showed excellent mechanical properties and bioactivity^14^. The mechanically strong CSi-Mg porous scaffolds were successfully fabricated via a bioceramic ink-writing technique for the highly efficient regeneration and repair of femoral or calvarial defects *in situ* in rabbit models. It was demonstrated that the surface bioactivity of CSi-Mg also benefited osteogenic cell proliferation and osteogenic gene expression in comparison with the clinically available β-TCP material^15,16^.

As the ZCC is a transitional interface, some researchers incorporated inorganic bioceramics (bone-phase material) into alginate/collagen hydrogel (cartilage-phase material) to mimic its composition. Alginate has been utilized extensively for chondrocyte culture and cartilage tissue engineering^17^. Chondrocytes have been shown to maintain their native morphology and produce proteoglycan and a collagen rich matrix in alginate, which also has the merit of being biocompatible, nonimmunogenic, and biodegradable. Type I collagen is one of the most abundant compositions in human tissues. The biocompatibility and controllable biodegradability of type I collagen make it fit for bone and cartilage tissue engineering^18^.

Herein, for the first time, we introduced novel CSi-Mg10 into osteochondral interface tissue-engineering scaffold fabrication. The objective of this study is to evaluate the potential effects of the novel 3D-printed hydrogel/CSi-Mg10 hybrid scaffolds on ZCC formation via a human deep zone chondrocyte (hDZC) culture experiment *in vitro*. It is hypothesized that the scaffolds containing 10% Mg-substituted CSi (hereby denoted CSi-Mg10) could more effectively induce the formation of ZCC *in vitro* than could the control hydrogel (without CSi-Mg10) or those containing nHAp or β-TCP.

## Results

### Characterization of inorganic powders and porous scaffolds

**Figure 1:**
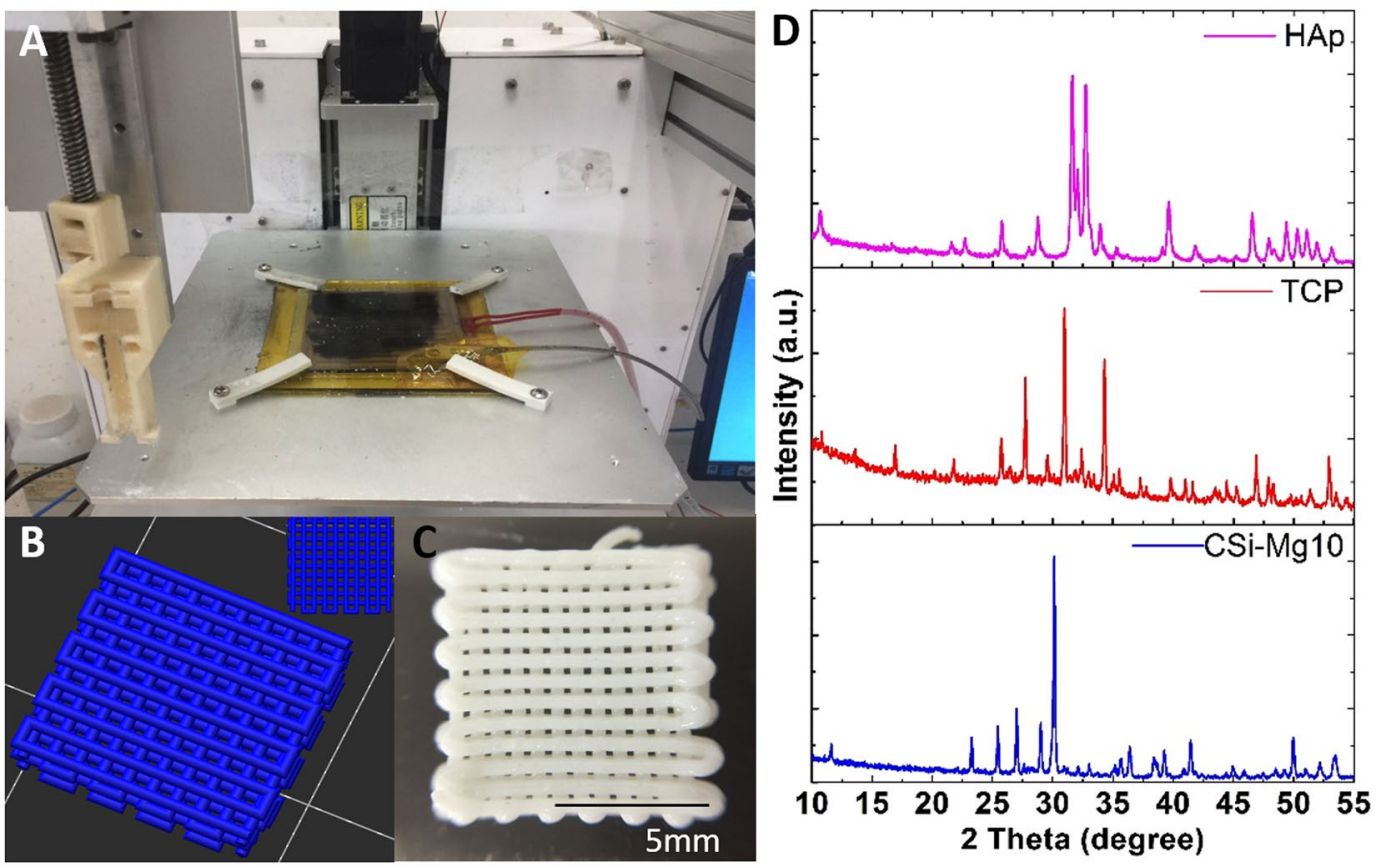
Characterization of nHAp, β-TCP and CSi–Mg10 ceramic powders and hydrogel-based scaffolds. (**A**) Photograph of 3D bioprinter. (**B**) The 3D model of the designed scaffold. (3D view, top view) (**C**) The outward appearance of the scaffold. (**D**) XRD patterns for the ceramic powders. Scale bars represent 5 mm.

The CSi-Mg/hydrogel hybrid scaffolds were fabricated with a bioink writing 3D printing system (Fig. 1A). Based on the 3D model of the designed scaffold (3D view, top-view in Fig. 1B), examination of the macroscopic appearance and morphology revealed that the as-printed scaffolds (9.1 mm × 9.1 mm) have vertically connected pores, with a strut diameter of ~450 µm and pore size of ~230 × 230 µm (Fig. 1C). In addition, the X-ray diffraction (XRD) patterns of bioceramic powders (CSi-Mg10, β-TCP, nHAp) presented in Fig. 1D confirmed that the β-TCP and nHAp powders were highly crystalline Ca-phosphates, while the CSi-Mg10 powders exhibited the pure wollastonite phase.

### Cell proliferation

**Figure 2:**
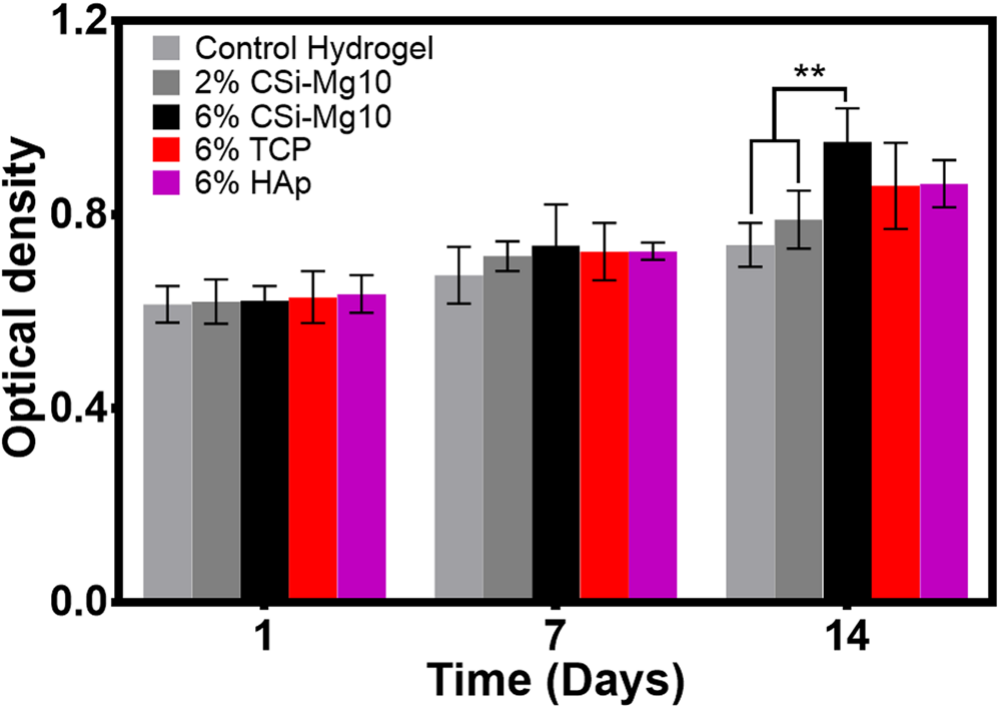
Cell proliferation of chondrocytes on (0–6)% w/v CSi-Mg10, β-TCP, and nHAp scaffolds. MTT assay at days 1, 7 and 14 for the control hydrogel, 2% and 6% CSi-Mg10, 6% β-TCP, 6% nHAp groups (**p < 0.01, n = 6).

Figure 2 shows that the chondrocytes remained viable and cell number increased for all groups over time. The overall cell viability of the four groups was higher than that of the control hydrogel group. Particularly, by day 14, it was seen that a significantly higher number of cells was found on the 6% CSi-Mg10 scaffolds than on the 2% CSi-Mg10 and control hydrogel scaffolds.

However, no significant difference was detected between the 6% CSi-Mg10, β-TCP and nHAp groups at all timepoints.

### GAG deposition

**Figure 3:**
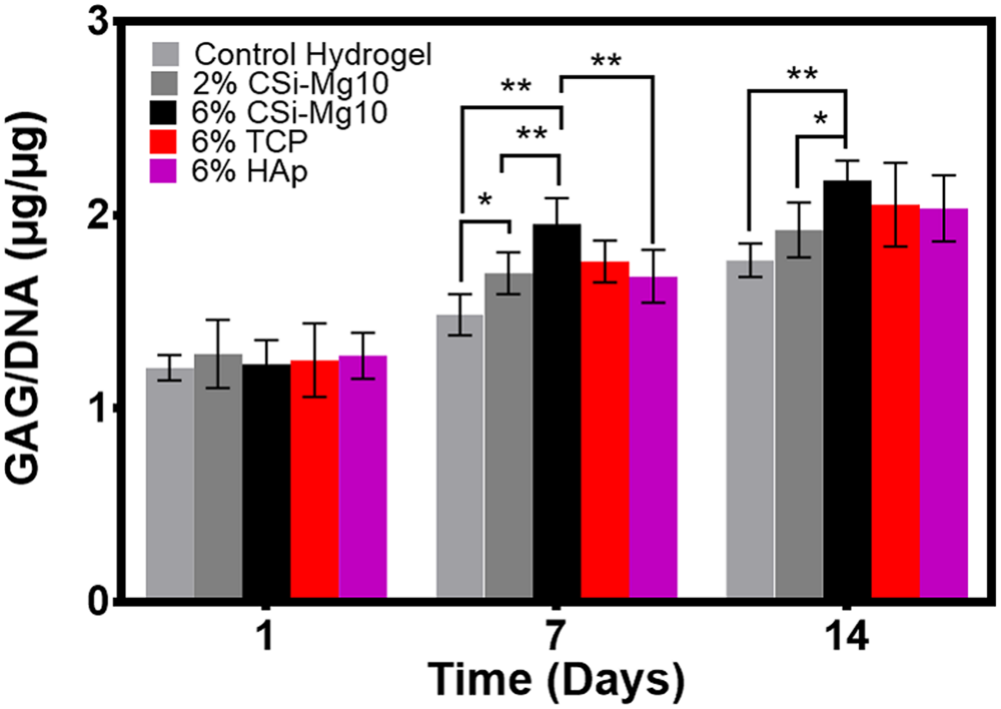
GAG deposition. GAG content at days 1, 7 and 14 for the control hydrogel, 2% and 6% CSi-Mg10, 6% β-TCP, and 6% nHAp groups (*p < 0.05, **p < 0.01, n = 6).

GAG/DNA was measured at different time stages (Fig. 3). A significant difference in GAG/DNA was measured at day 7 in the 6% CSi-Mg10 scaffold group compared with that of the 2% CSi-Mg10 scaffolds and the control hydrogel (*p* < 0.01). At day 14, the GAG/DNA maintained highest in 6% CSi-Mg10 scaffold groups compared with control hydrogel (*p* < 0.01) and 2% CSi-Mg10 scaffold (*p* < 0.05), while the difference between the 2% and 6% CSi-Mg10 groups was reduced.

At day 7, a significant difference was observed between the 6% CSi-Mg10 and nHAp group (*p* < 0.01). However, no significant difference was detected between the 6% CSi-Mg10, β-TCP and nHAp groups at day 14.

### Immunohistochemistry Analysis

**Figure 4:**
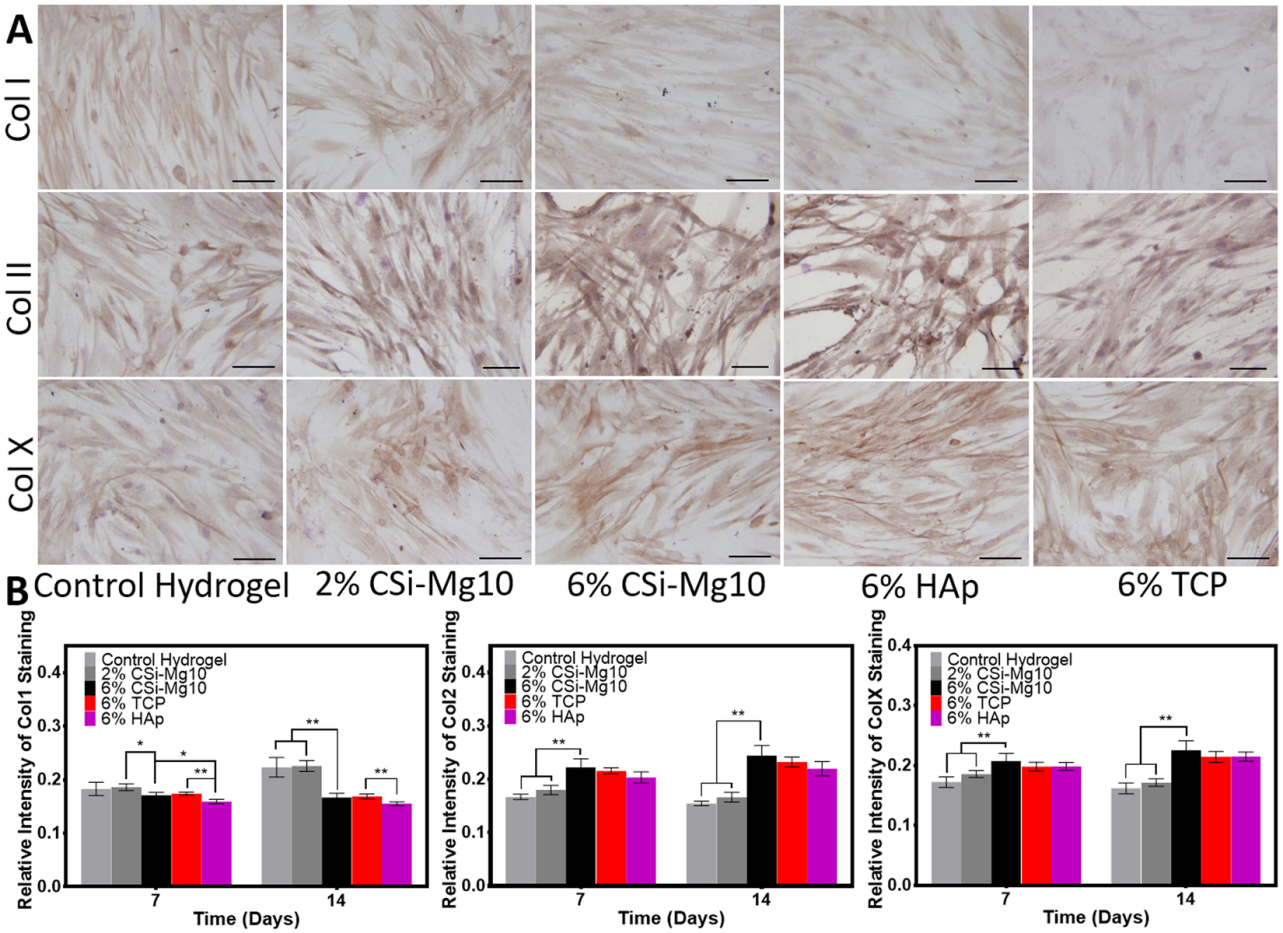
Immunohistochemistry. (**A**) Immunohistochemistry staining for collagen type I, II and X at day 7 for control hydrogel, 2% and 6% CSi-Mg10, 6% β-TCP, and 6% nHAp groups and (**B**) quantitative analysis at days 7 and 14 (*p < 0.05, **p < 0.01, n = 6). The black bar represents 50 μm.

To evaluate the specific osteogenic, chondrogenic or hypertrophic chondrogenic ECM protein zones within the scaffold, an immunohistochemistry technique was used to analyze type I collagen, type II collagen and type X collagen at days 7 and 14 as shown in Fig. 4. A significantly lower collagen type I deposition was found in the 6% CSi-Mg10 scaffolds, along with significantly higher collagen type II and collagen type X deposition, compared with the depositions found in the control hydrogel and the 2% CSi-Mg10 scaffold. Furthermore, the difference increased as time progressed.

Immunohistochemistry staining also showed that the collagen type I deposition was lowest in the nHAp group, with a significant difference at day 7. The collagen type II and type X deposition was similar and did not show any significant difference when comparing the 6% CSi-Mg10 scaffold with the 6% β-TCP and nHAp scaffolds at days 7 or 14.

### Mineralization and hypertrophy

**Figure 5:**
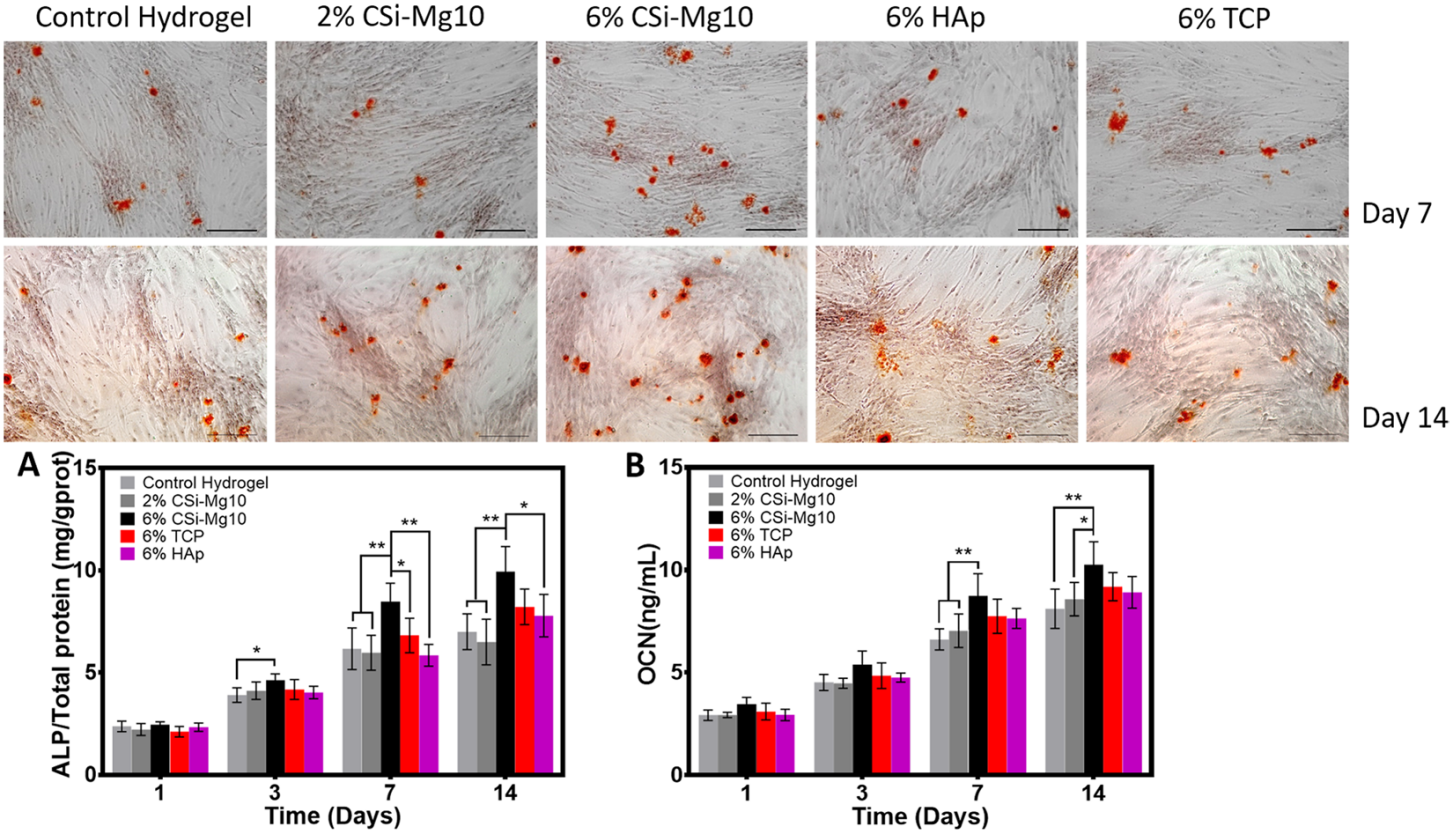
Mineralization and hypertrophy. Alizarin Red S staining for the control hydrogel, 2% and 6% CSi-Mg10, 6% β-TCP, and 6% nHAp groups, respectively, at day 7 and 14 (n = 3). (**A**) Cell ALP activity analysis at day 1, 3, 7 and 14 (*p < 0.05, **p < 0.01, n = 6). (**B**) Cell osteocalcin analysis at days 1, 3, 7 and 14 (*p < 0.05, **p < 0.01, n = 6). The black bar represents 100 μm.

For mineralization deposition tests, Alizarin Red S staining was performed at day 7 and 14. As seen from Fig. 5, calcium nodules appeared in all groups at day 7 and 14. Compared with the calcium nodules and staining intensity in the control hydrogel and 2% CSi-Mg10 groups, those in the 6% CSi-Mg10 group increased significantly, while the maximum calcium nodule appearance was found in the 6% CSi-Mg10 group compared with that in the 6% β-TCP and nHAp groups at both day 7 and 14.

Chondrocyte ALP activity was measured at day 1, 3, 7 and 14 as shown in Fig. 5A. A significant increase in ALP activity was observed over time in all groups (*p* < 0.05). At day 3, a significant difference in ALP activity was detected in the 6% CSi-Mg10 group compared with that in the control hydrogel (*p* < 0.05). At days 7 and 14, the 6% CSi-Mg10 scaffold group measured the highest ALP activity among all the other groups with significant difference, except when compared with the activity of the β-TCP scaffold group at day 14. At days 7 and 14, no significant difference was found between either the control hydrogel and the 2% CSi-Mg10 group or the β-TCP and the nHAp scaffold group.

An osteocalcin (OC) assay was also performed to evaluate mineralization and hypertrophy (Fig. 5B). The trend was generally similar to that of the ALP activity. The OC content in the 6% CSi-Mg10 scaffold was highest compared with that of the control hydrogel and the 2% CSi-Mg10 scaffold at both days 7 and 14; it was also higher than that of the β-TCP and the nHAp scaffolds, although the difference was not found to be significant.

### Western blot for protein expression

**Figure 6:**
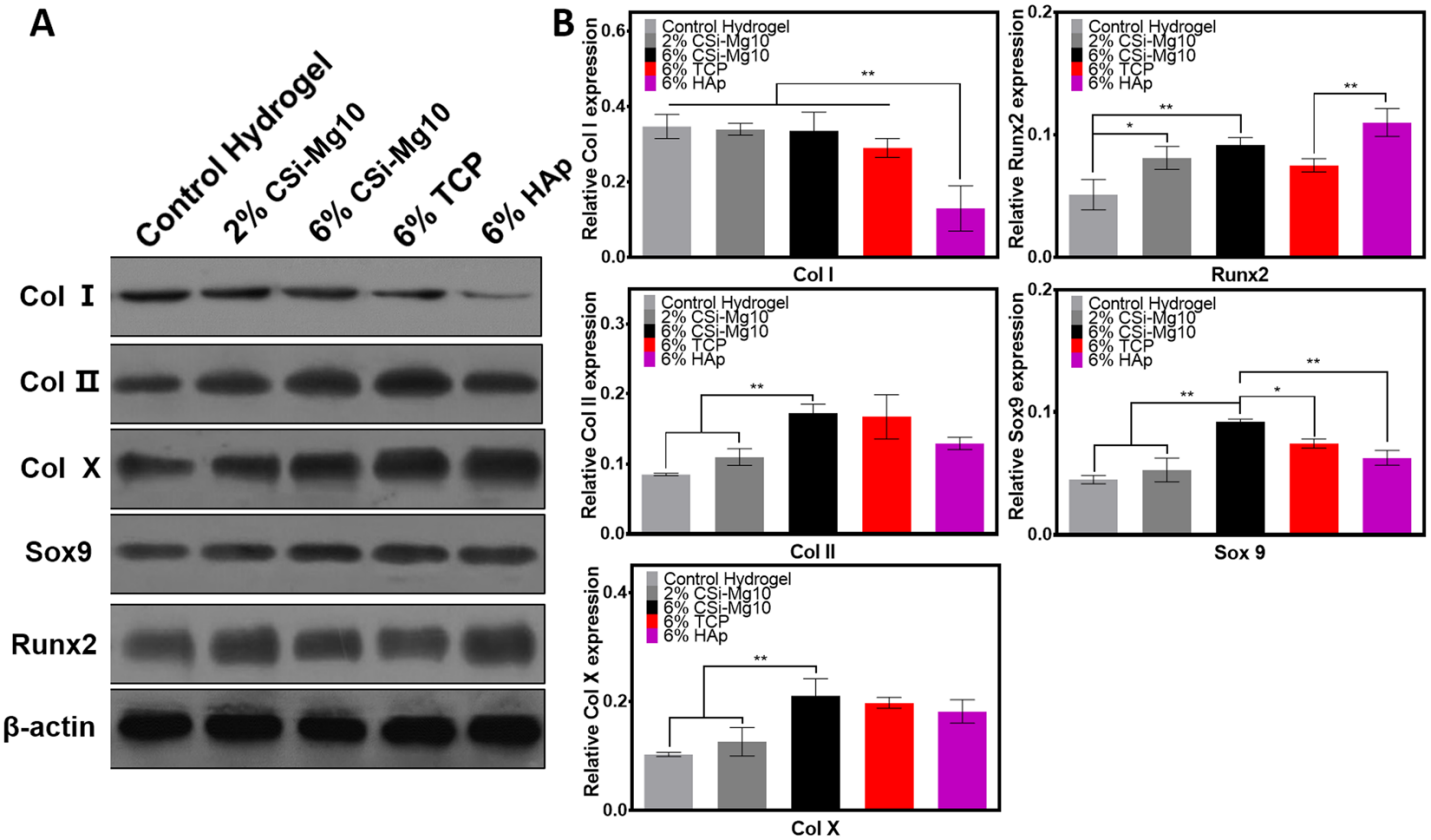
Western blot. (**A**) Specific protein expression by western blot at day 14 for the control hydrogel, 2% and 6% CSi-Mg10, 6% β-TCP, and 6% nHAp groups. (**B**) Quantitative analysis of western blot for osteogenic, chondrogenic and hypertrophic protein expression levels at day 14 (n = 3).

The effect of biomaterials on transcription of osteogenic, chondrogenic and hypertrophic chondrogenic protein expression at day 14 was examined in this study (Fig. 6). Among all groups, the expression of collagen type I was lowest in the nHAp scaffold (*p* < 0.01). The expression levels of collagen type II, type X, Runx2 and Sox-9 were increased with increasing CSi-Mg10 percentage.

Additionally, the expression of Sox-9 in the 6% CSi-Mg10 scaffold was significantly higher than that in the β-TCP (*p* < 0.05) and nHAp scaffolds (*p* < 0.01). The expression of Runx2 was significantly higher in nHAp than that in β-TCP (*p* < 0.01).

## Discussion

In this study, a CSi-Mg10 bioceramic-hydrogel was combined with aerosol crosslinking and additive manufacturing techniques to successfully fabricate bioactive porous scaffolds for the first time. Effects of different types of scaffolds on chondrocyte mineralization, hypertrophy and matrix deposition were evaluated *in vitro*. The results demonstrated that all biomaterials are biocompatible for chondrocytes. An increase in the CSi-Mg10 dose in the scaffolds was followed by enhanced mineralization and GAG production, with elevated expression of hypertrophic and chondrogenic markers for chondrocytes. In particular, enhanced mineralization without elevated GAG production for the 6% CSi-Mg10 scaffold group was noted while comparing with frequently used Ca-phosphate biomaterials in clinic. The 6% CSi-Mg10 scaffold was therefore assumed to be optimal, as the hypertrophic chondrocytes balanced the matrix and the mineral production to meet with the description of calcified cartilage.

Hypertrophic chondrocytes in the cartilage are found to express OC during the mineralized state, such as in endochondral ossification^19^. Pullig *et al*. reported that the expression of OC in chondrocytes correlates with chondrocyte hypertrophy^20^. OC is considered a specific indicator of osteoblast activity and is involved in bone formation^21^. The upregulation of OC in our study indicates the hypertrophy and subsequent mineral production of chondrocytes. GAG has been reported as one of the utmost important matrix constituents in cartilage^22^, and the osteochondral interface has collagen-rich ECM, which mainly includes types I, II, and X collagens^23^. Interestingly, collagen types II and X were upregulated, while collagen type I was unchanged in western blot for the 6% CSi-Mg10 scaffold group. The higher expression of collagen type II is a reflection of both secretion stimulation as well as the cartilaginous, instead of osseous, resemblance of ECM^24^. Sox-9 is a member of the SOX (Sry-related high mobility group box) family of transcription factors. The expression of Sox-9 can be detected in progenitor cells in many different organs^25^ with chondrocytes excluded. In our study, expression of Sox-9 was upregulated with the addition and increasing dose of CSi-Mg. As was observed during endochondral ossification, the expression of Sox-9 starts in mesenchymal progenitor cells, remains high in chondrocytes, and ceases in prehypertrophic chondrocytes^26,27^. According to Daisuke *et al*., the deletion of Sox-9 in flat chondrocytes caused the absence of hypertrophic chondrocytes^28^. These suggests that the expression of Sox-9 is essential for chondrocyte hypertrophy and necessary for the appropriate subsequent endochondral bone formation to chondrocytes and the survival of hypertrophic chondrocytes. Mineralization was thought to be an indicator of the formation of the osteochondral interface^29^. In our study, it was observed that the response of hDZCs to CSi-Mg10 was dose-dependent. Chondrocyte ALP activity and precipitation of calcium nodule were found to be the highest in the scaffolds with 6% CSi-Mg10. Taken together, chondrocyte hypertrophy (collagen type X, Sox-9 and OC), elevated collagen depositions and expressions (as shown in immunohistochemistry staining and western blot) and mineralization are indicators of a calcified cartilage matrix formation, since collagen regulates the size and shape of mineral crystals and mineral deposition^30^.

In fact, numerous studies have paid attention to Ca-silicate (CSi) ceramics due to their high bioactivity *in vitro* and *in vivo* over the past two decades^31^. These bioceramics are superior to calcium phosphates (CaPs) in cell attachment, proliferation and differentiation^32^. The positive effects resulting from the release of some elements (ions), such as calcium and silicon from CSi, on their surrounding biological environment have been widely demonstrated^33,34^. Fiocco and colleagues confirmed that the biocompatibility of Ca-Mg silicate ceramics and the osteogenic differentiation were enhanced by Mg addition^35^. It has been reported that magnesium ions (Mg^2+^) enhance bone regeneration by promoting the proliferation and differentiation of osteogenic (stem) cells via osteogenesis signaling pathways *in vitro*^36,37^. Additionally, Landi *et al*. found that Mg-doped HAp showed greater bioconductivity than that of pure HAp^38^. Our study also demonstrated the superiority of the 6% CSi-Mg10 scaffolds over conventional Ca-phosphate materials. For the two Ca-phosphate materials (β-TCP and nHAp), the properties of both were mostly similar, but higher collagen type I deposition and expression was found in the β-TCP scaffold group. This result is in line with the observation of Rojbani *et al*. that at 8 weeks, the new bone formation was significantly higher in the β-TCP group than that in the HAp group^39^. Previous studies have shown that cartilage mineralization is proceeded by accumulation of some critical mineral elements such as calcium ions and phosphate groups^40,41^. As mineral deposits in calcified cartilage are often associated with collagen fibers, the elevated collagen deposition may facilitate cell-mediated mineralization.

In our study, DZC was selected as source of cells. Khanarian *et al*. compared the response of full-thickness chondrocytes with that of DZC in alginate and alginate + HAp scaffolds and found that DZC produced more collagen and proteoglycan and had higher ALP activity than did full-thickness chondrocytes^24^. It is noted that the expression level of the hypertrophic marker and mineralization potential were increased when DZCs were cultured in alginate + HA scaffolds^24^. In addition, surface and middle-zone chondrocytes seeded in hydrogel scaffolds with or without HA only had basal ALP activity over time. The above indicates that DZC-like cells seeded in the composite scaffold is a rational approach.

Cartilage regeneration is a multifaceted and elusive process. In the case of articular cartilage lesions, clinically available cell-based tissue engineered scaffolds such as Hyalograft^®^ C, NeoCart^®^, NOVOCART^®^ 3D, INSTRUCT and (M)ACI techniques have achieved good outcomes. However, these scaffolds are not capable of subchondral bone repair. The treatment becomes more challenging when the chondral lesion is larger and subchondral bone is involved. The dysfunction of zone of calcified cartilage impairs the process of osteochondral repair.

In view of the above concerns, triphasic scaffolds (gradual change in ratio between collagen type I and HA in each layer) known as MaioRegen^®^ were investigated, where the orderly osteochondral tissue was regenerated with formation of hyaline-like cartilage^42^. It is envisioned that when our scaffold is utilized together with hydrogel and ceramic scaffolds as chondral and bony phase scaffold respectively, it might simultaneously promote chondrogenic differentiation in chondral layer, osteogenic differentiation in subchondral bone, and reconstruction of calcified layer in the middle. Additionally, the autologous DZCs can be harvested from cartilages in non-weightbearing area of knee via arthroscopic biopsy and co-cultured with scaffolds.

There are some limitations in this study. The osteochondral interface is one of the most complex tissues to regenerate due to its small size and high degree of heterogeneity. On one hand, the mechanical evaluation of the scaffolds is still insufficient, and improved and delicate processing methods of the scaffolds must be explored. On the other hand, future studies will focus on evaluating the effect of adding triiodothyronine (T3) during cell culture to enhance hypertrophy. Biological outcomes of such scaffolds *in vivo* with a long-term goal of achieving functional and integrative OCD repair will be investigated.

## Conclusion

This study incorporated CSi-Mg10 to fabricate bioactive composite scaffolds aiming to regenerate ZCC for the first time. The scaffolds containing 6% CSi-Mg10 were optimal for the formation of a calcified cartilage-like matrix *in vitro* and are promising for osteochondral interface tissue engineering.

## Materials and Methods

### Preparation of inorganic powders

CSi–Mg10 powders with 10 mol% Ca substituted by Mg were synthesized by a wet-chemical precipitation method as described previously^43^. The powders were ground in a planetary ball miller to a particle size of below 5 μm. β-TCP powders were synthesized as reported^44^ and then ground to a particle size of below 5 μm. nHAp powders were purchased from Sinopharm Reagent Co., Ltd. The phase composition of the powders was confirmed by XRD (Rigaku Co., Akishima, Japan).

### 3D-printing scaffolds

Sodium alginate was dissolved in distilled water at a concentration of 4% (w/v), collagen type I at a concentration of 2% (w/v) and inorganic superfine powders (control hydrogel, 2% CSi-Mg10, 6% CSi-Mg10, 6% β-TCP and 6% nHAp) were added under stirring until a homogeneous solution with a suitable viscosity for 3D-printing was achieved. The 3D scaffolds were fabricated using 3D direct bioceramic/hydrogel ink writing equipment (with a homemade precision three-axis positioning system and an extruding device derived by the step motor, which is mounted on the *x*-axis). For layer-by-layer writing, the ink was added to a 1 mL syringe and extruded through a conical nozzle by moving a piston rod. An aerosol humidifier (SKEEN; China) containing a CaCl_2_ solution (5% (w/v)) was used to solidify the structure every time a layer was printed. The nozzle diameter was 400 μm, and the moving speed of the dispensing unit was set to 6 mm s^−1^. After printing and primary crosslinking treatment, the 3D structure was then further cross-linked with a CaCl_2_ solution (5% (w/v)) for 5 min. The scaffolds (*denoted* control hydrogel, 2% CSi-Mg, 6% CSi-Mg, 6% β-TCP, and 6% nHAp) were washed with PBS (Shanghai Long Island antibody diagnostic reagents company (FL-2004), Shanghai, China) and were exposed to ^60^Co radiation for sterilization before subsequent *in vitro* cell seeding.

### Cell culture *in vitro*

Primary articular chondrocytes were isolated from human knee joints after patients signed an informed consent form. All methods were carried out in accordance with the relevant guidelines and regulations of the 2^nd^ Affiliated Hospital, School of Medicine, Zhejiang University, and all experimental protocols were approved by the 2^nd^ Affiliated Hospital, School of Medicine, Zhejiang University Ethics Committee. Cells that were digested from the bottom third of articular cartilage (removing ZCC) were designated hDZCs^45^. The cartilage fragments were digested for 16 h with type II collagenase (Sigma) dissolved in Dulbecco’s modified Eagle’s medium (DMEM; HyClone, Utah, USA) containing 10% fetal bovine serum (FBS; HyClone, Utah, USA), 2% antibiotics (10,000 U/mL penicillin and 10 mg/mL streptomycin), and 0.2% antifungal (amphotericin B). The cell suspension was then filtered with a 30 μm filter before plating. The isolated chondrocytes were incubated in high-density culture (1 × 10^6^/mL) in DMEM supplemented with 10% FBS.

### Cell proliferation and GAG deposition

MTT assay (MTT, Sigma-Aldrich) was used to evaluate the cytocompatibility of the 3D-printed organic/inorganic hybrid scaffolds (control hydrogel, 2% CSi-Mg, 6% CSi-Mg, 6% β-TCP, and 6% nHAp; n = 6). Briefly, the concentration of hDZCs was adjusted to 1 × 10^6^ cells/mL, and hDZCs were seeded into the different scaffolds and incubated at 37 °C, with 5% CO_2_ for 1, 3, 7 and 14 days. Then, 10 μL MTT was added to each well, and the plate was incubated at 37 °C under 5% CO_2_ in the cell incubator for 3–4 h away from light. Then, 200 μL of DMSO was added after fluid was aspirated from the well, and the plate was shaken with a shaker (Aohua, Changzhou, China) for 10 min. The absorbance of the culture media was measured at 492 nm using a Microplate Reader (Thermo Scientific, USA).

Sample glycosaminoglycan (GAG) content (n = 6) was determined with a modified 1,9-dimethylmethylene blue (DMMB) binding assay, with chondroitin-6-sulfate (Sigma-Aldrich) as the standard, at days 1, 7 and 14. Briefly, the cells were digested with 0.25% trypsin (Solarbio, Beijing, China). The cell suspension was centrifuged at 1000 rad/min for 10 min in a 10 mL centrifuge tube. The precipitate was collected and was put into 1 mL of papain digestion solution and digested at 60 °C for 16 h. The samples were centrifuged at 1000 rad/min for 10 min, 100 μL of supernatant was collected and 2.5 mL of DMMB staining liquid was added in. The absorbance was measured at 525 nm and recorded with spectrophotometer. To normalize GAG, DNA content was quantified with DNA Quantification Kit (Solarbio) according to the manufacturer’s instruction.

### Mineralization and hypertrophy

Alkaline phosphatase (ALP) activity (n = 6) of the hDZCs was evaluated by an assay reagent kit (Nanjing Jiancheng Bioengineering Institute, Jiangsu, China). Briefly, at days 1, 3, 7 and 14, the samples were lysed in 0.1% Triton X-100 solution (Beyotime (ST795), Shanghai, China), and after incubation, the lysate was transferred and incubated in a water bath for 15 min at 37 °C. The plate was shaken gently, and the OD value of each well was measured at the wavelength of 520 nm.

Osteocalcin (OC) detection with ELISA (control hydrogel, 2% CSi-Mg, 6% CSi-Mg, 6% β-TCP, 6% nHAp; n = 6) was performed at day 1, 3, 7 and 14. Briefly, hDZCs were seeded on scaffolds and incubated at 37 °C, with 5% CO_2_. The supernatants of the cells were collected and OC levels were detected with an ELISA kit for OC (You’er Shengmao (SEA471Hu), Wuhan, China) according to the manufacturer’s instructions at appropriate timepoints respectively.

### Western blotting

At day 14, western blot analysis (n = 3) was conducted to analyze the relative expression level of collagen types I, II, X, Sox-9 and Runx2 in the hDZCs. The lysis buffer (Radio-Immunoprecipitation Assay buffer, Beyotime, Shanghai, China) was added (100 μL), and the proteins were transferred to nitrocellulose membranes (Millipore, Darmstadt, Germany). The membranes were put into plates, and a blocking solution containing 5% skim milk powder at room temperature was added. The plate was then shaken with oscillations for 1.5–2 h, after which the membranes were placed in plates containing the primary antibody dilution solution and shaken at 4 °C overnight; subsequently, the plates with the membranes were incubated with goat anti-rabbit HRP-labeled secondary antibody (A0208, Beyotime, Shanghai, China) for 1 h at room temperature. Anti-β-actin antibody was used as a protein loading control. Quantitative analysis was performed using a Gel Pro analyzer 6.0 (Media Cybernatics, USA) and was shown as the relative expression to β-actin.

### Alizarin Red S staining and immunohistochemistry staining

Alizarin Red S staining (n = 3) was performed using an Alizarin Red Detection Kit (Fiveheart Inc., Xi’an, China). After 7 and 14 days of culturing, 1 mL of 4% paraformaldehyde (80096618, Sinopharm Group, China) solution was added to each well, and the plate was incubated at 4 °C for 15 min. The supernatant was discarded, the wells were washed 3 times with PBS, and 500 μL Alizarin Red dye staining solution was added to each well. The plate was incubated at room temperature for 30 min. The calcium nodule formation was observed using an inverted microscope (Leica, Germany).

At days 7 and 14 immunohistological staining was used to investigate the extracellular matrix (ECM) proteins, including collagen types I, II and X, which were produced by the hDZCs (n = 6). After fixation, samples were treated with 1% hyaluronidase for 30 min at 37 °C to remove proteoglycan and then were incubated with primary antibody overnight. The images of the slices were taken on an optical microscope (BX41, Olympus, Japan). Quantitative analysis was performed using Image Pro plus 6.0 software (Media Cybernatics, USA). Immunohistological staining was repeated for six times to calculate average staining intensity.

### Statistical analysis

All numerical data were expressed as the mean value ± standard deviation (SD), with n equal to the number of samples analyzed. Statistical analysis was performed with a one-way analysis of variance (one-way ANOVA), and *p* < 0.05 was considered to be significant. The Tukey HSD/Dunnett T3 *post hoc* test was used for all pairwise comparisons, and significance was attained at *p* < 0.05. All statistical analyses were performed using the SPSS 20.0 software (SPSS, Chicago, IL, USA). *p* < 0.05 was considered to be significant and is indicated by *. *p* < 0.01 is indicated by **.

## Electronic supplementary material

Supplementary Information
