# Supplementary material for: In vitro Chondrocyte Responses in Mg-doped Wollastonite/Hydrogel Composite Scaffolds for Osteochondral Interface Regeneration
Source: Sci Rep. 2018 Dec 17;8:17911. doi: 10.1038/s41598-018-36200-x (PMC6297151; doi:10.1038/s41598-018-36200-x)

In vitro Chondrocyte Responses in Mg-doped Wollastonite/Hydrogel Composite Scaffolds for Osteochondral Interface Regeneration

Xinning Yu[1,2,4], Tengfei Zhao[1,2], Yiying Qi[1,2], Jianyang Luo[1,2], Jinghua Fang[1,2,4], Xianyan Yang[3], Xiaonan Liu[1,2], Tengjing Xu[1,2], Quanming Yang[1,2], Zhongru Gou[3],Xuesong Dai[1,2*]

[1]Department of Orthopaedic Surgery, Second Affiliated Hospital, School of Medicine, Zhejiang University, Hangzhou 310009, China.

[2]Orthopaedics Research Institute, Zhejiang University, Hangzhou, 310009, China.

[3]Bio-nanomaterials and Regenerative Medicine Research Division, Zhejiang-California International NanoSystems Institute, Zhejiang University, Hangzhou 310058, China.

[4]Department of Orthopaedic Surgery, Hangzhou Mingzhou Hospital, Hangzhou 311215, China.

Correspondence should be addressed to Xuesong Dai (E-mail: daixshz@zju.edu.cn)

Figure S1

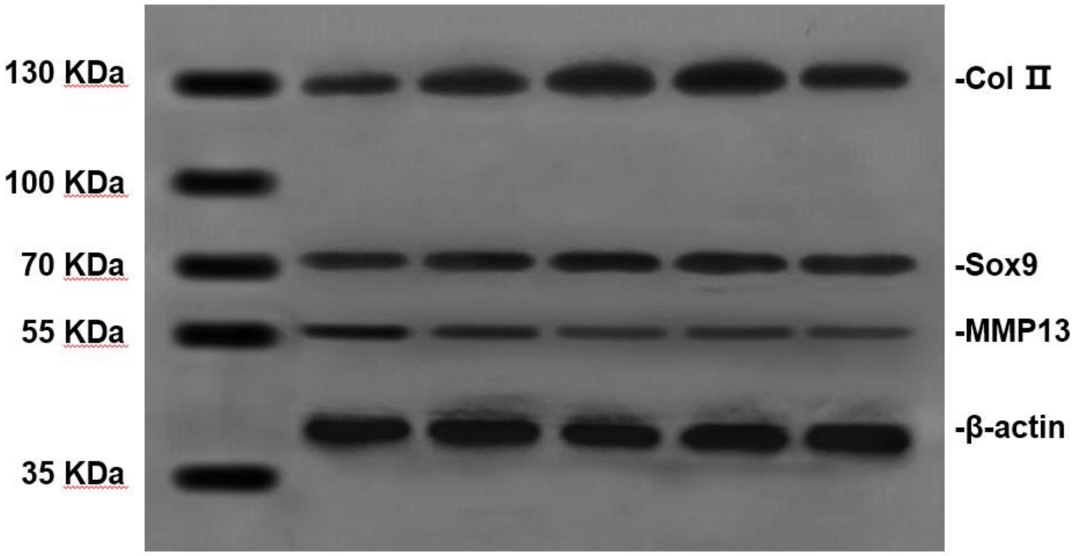

Figure S2

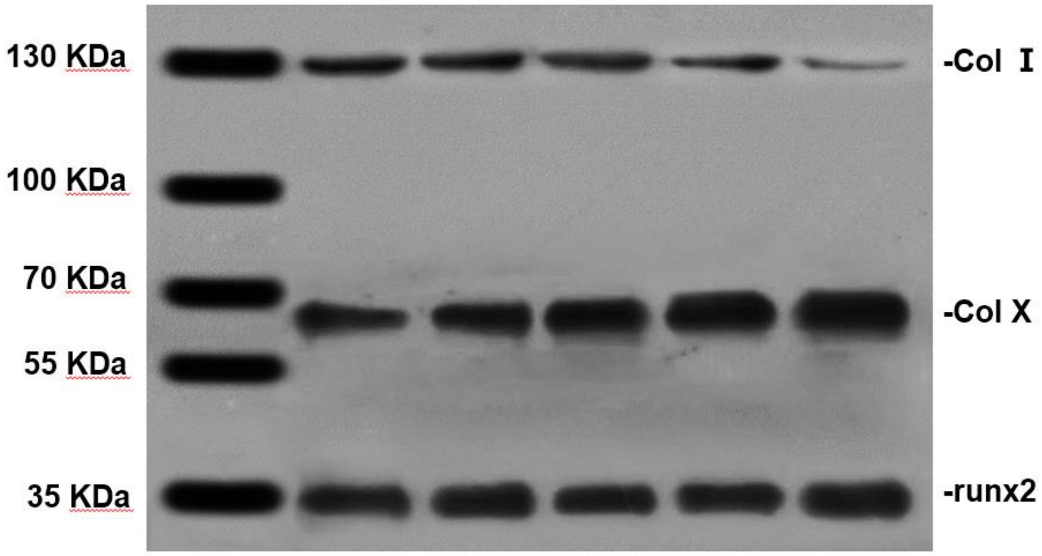

Figure S3

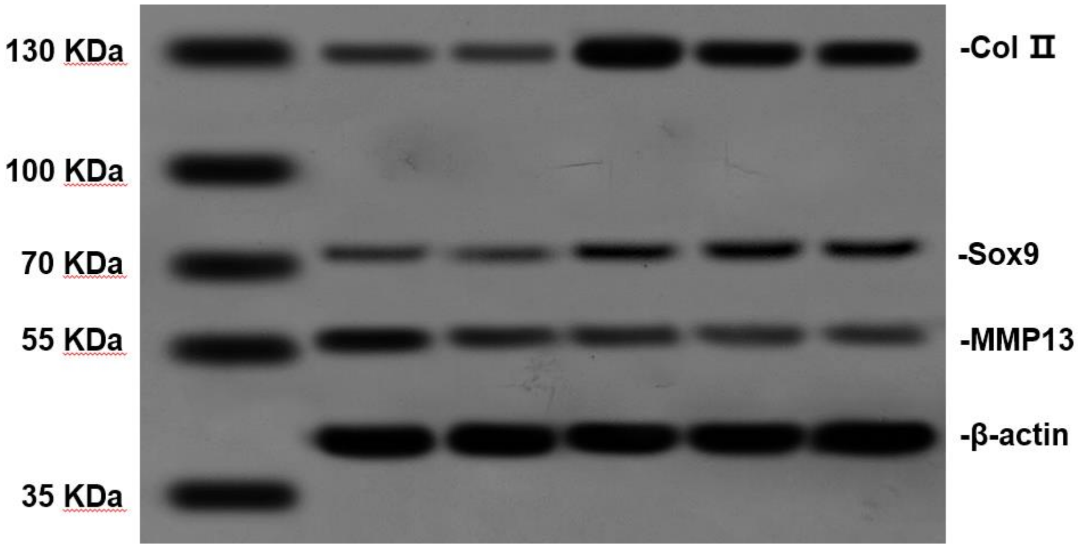

Figure S4

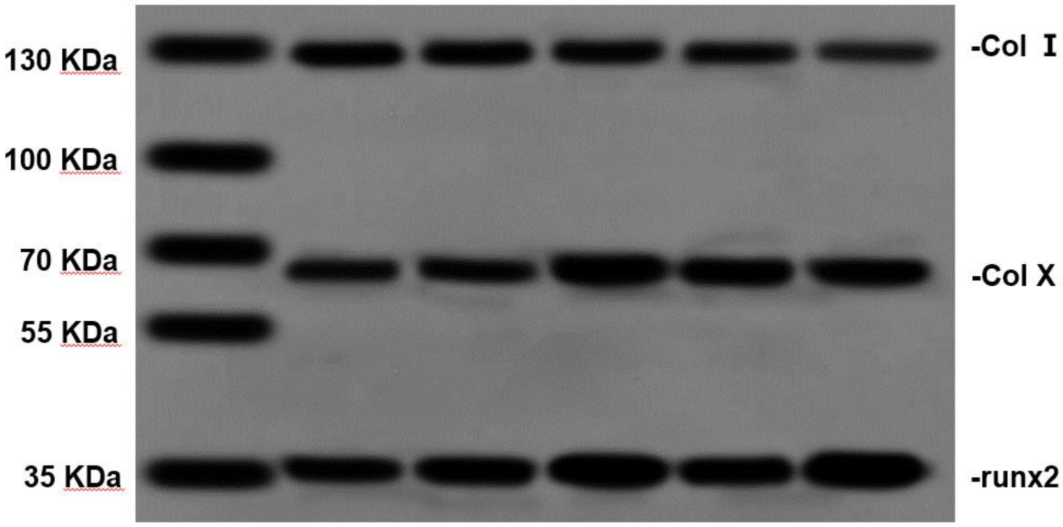

Figure S5

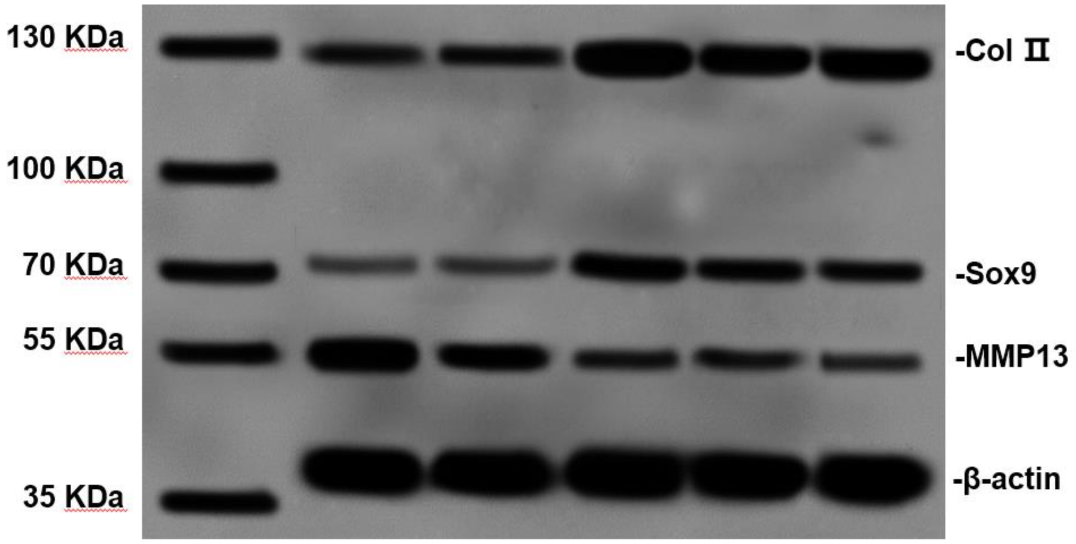

Figure S6

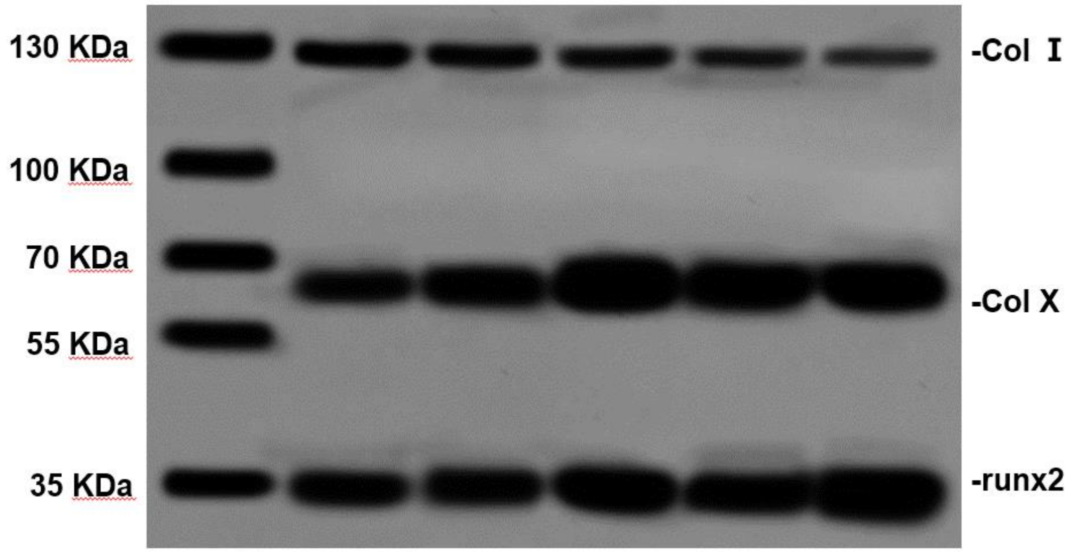

Supplement: Supplementary file 1 — Supplementary Information [file 41598_2018_36200_MOESM1_ESM.pdf]
